# Spatial patterns of progressive brain volume loss after moderate-severe traumatic brain injury

**DOI:** 10.1093/brain/awx354

**Published:** 2018-01-04

**Authors:** James H Cole, Amy Jolly, Sara de Simoni, Niall Bourke, Maneesh C Patel, Gregory Scott, David J Sharp

**Affiliations:** Computational, Cognitive and Clinical Neuroimaging Laboratory, Imperial College London, Division of Brain Sciences, Hammersmith Hospital, London, UK

**Keywords:** traumatic brain injury, voxel-based morphometry, longitudinal, neurodegeneration, brain atrophy

## Abstract

Traumatic brain injury leads to significant loss of brain volume, which continues into the chronic stage. This can be sensitively measured using volumetric analysis of MRI. Here we: (i) investigated longitudinal patterns of brain atrophy; (ii) tested whether atrophy is greatest in sulcal cortical regions; and (iii) showed how atrophy could be used to power intervention trials aimed at slowing neurodegeneration. In 61 patients with moderate-severe traumatic brain injury (mean age = 41.55 years ± 12.77) and 32 healthy controls (mean age = 34.22 years ± 10.29), cross-sectional and longitudinal (1-year follow-up) brain structure was assessed using voxel-based morphometry on T_1_-weighted scans. Longitudinal brain volume changes were characterized using a novel neuroimaging analysis pipeline that generates a Jacobian determinant metric, reflecting spatial warping between baseline and follow-up scans. Jacobian determinant values were summarized regionally and compared with clinical and neuropsychological measures. Patients with traumatic brain injury showed lower grey and white matter volume in multiple brain regions compared to controls at baseline. Atrophy over 1 year was pronounced following traumatic brain injury. Patients with traumatic brain injury lost a mean (± standard deviation) of 1.55% ± 2.19 of grey matter volume per year, 1.49% ± 2.20 of white matter volume or 1.51% ± 1.60 of whole brain volume. Healthy controls lost 0.55% ± 1.13 of grey matter volume and gained 0.26% ± 1.11 of white matter volume; equating to a 0.22% ± 0.83 reduction in whole brain volume. Atrophy was greatest in white matter, where the majority (84%) of regions were affected. This effect was independent of and substantially greater than that of ageing. Increased atrophy was also seen in cortical sulci compared to gyri. There was no relationship between atrophy and time since injury or age at baseline. Atrophy rates were related to memory performance at the end of the follow-up period, as well as to changes in memory performance, prior to multiple comparison correction. In conclusion, traumatic brain injury results in progressive loss of brain tissue volume, which continues for many years post-injury. Atrophy is most prominent in the white matter, but is also more pronounced in cortical sulci compared to gyri. These findings suggest the Jacobian determinant provides a method of quantifying brain atrophy following a traumatic brain injury and is informative in determining the long-term neurodegenerative effects after injury. Power calculations indicate that Jacobian determinant images are an efficient surrogate marker in clinical trials of neuroprotective therapeutics.

## Teaser

Using longitudinal neuroimaging, Cole *et al.* show that traumatic brain injury (TBI) results in progressive loss of brain tissue that continues for years after an injury. Tissue loss occurs predominantly in cerebral white matter and cortical sulci. Neuroimaging may be a feasible method for evaluating neuroprotective therapies after TBI.

## Introduction

Traumatic brain injury (TBI) triggers progressive neurodegeneration and is a risk factor for various types of dementia (Wang *et al.*, 2012). Currently no treatments for modifying these progressive changes exist. Experimental brain injury produces volume loss that is substantial and progressive (Smith *et al.*, 1997). Lower brain volume, disrupted white matter structure, neuroinflammation and increased age-related atrophy are seen in human studies (Blatter *et al.*, 1997; Kinnunen *et al.*, 2011; Ramlackhansingh *et al.*, 2011; Sharp and Ham, 2011; Cole *et al.*, 2015).

Atrophy is consistently observed on neuroimaging in the months and years after TBI (Ross, 2011). This can be sensitively identified using volumetric MRI measures. Previous studies have demonstrated this using either voxel-based morphometry (Bendlin *et al.*, 2008; Sidaros *et al.*, 2009; Farbota *et al.*, 2012) or summary measures from regions of interest (Ng *et al.*, 2008; Warner *et al.*, 2010; Ross *et al.*, 2012; Tomaiuolo *et al.*, 2012; Zhou *et al.*, 2013; Brezova *et al.*, 2014; Green *et al.*, 2014). This progressive neurodegeneration likely relates to the risk of long-term cognitive decline and dementia. Potentially, atrophy measurements could be used in clinical trials as surrogate outcomes of neuroprotective therapy efficacy, under the assumption that slowing atrophy will delay the onset of functional decline and disease. However, the existing data on atrophy measurements in TBI patients have key limitations regarding experimental design and analysis methods, motivating further investigation of atrophy patterns.

Many studies have investigated atrophy rates in the acute and subacute phase after TBI, usually less than 2 months after injury (Bendlin *et al.*, 2008; Ding *et al.*, 2008; Sidaros *et al.*, 2008; Xu *et al.*, 2010; Zhou *et al.*, 2013). Such analyses can be confounded by the acute effects of injury, particularly oedema. Moreover, the early measurements do not provide information about the time course of neurodegeneration into the chronic phase, and so are less relevant for considering evaluations of long-term neuroprotective therapies. In addition, several studies have included small numbers of patients (e.g. <20) or had highly variable between-scan intervals, making the interpretation of between-subject variability difficult (MacKenzie *et al.*, 2002; Marcoux *et al.*, 2008; Ng *et al.*, 2008; Farbota *et al.*, 2012; Ross *et al.*, 2012; Tomaiuolo *et al.*, 2012; Green *et al.*, 2014). Crucially, most studies have lacked appropriate longitudinal control groups (Ding *et al.*, 2008; Marcoux *et al.*, 2008; Ng *et al.*, 2008; Warner *et al.*, 2010; Xu *et al.*, 2010; Ross *et al.*, 2012; Tomaiuolo *et al.*, 2012; Brezova *et al.*, 2014). This is essential for modelling normal age-related changes and corresponding measurement variability. Without this, changes in patients cannot be properly considered in the context of healthy ageing. Another issue is that the within-subject nature of a longitudinal design has not previously been properly accounted for (Bendlin *et al.*, 2008; Farbota *et al.*, 2012; Tomaiuolo *et al.*, 2012). This approach ignores the correlations between serial measures (Reuter *et al.*, 2012) and potentially biases estimates of atrophy (Kim *et al.*, 2013). Finally, most studies failed to explicitly address the important issue of focal lesions on atrophy measures, which have been shown to influence automated image processing procedures (Brett *et al.*, 2001).

Here we address these issues and lay out a framework for using atrophy measures in clinical trials, as is common in other neurodegenerative contexts (Cash *et al.*, 2014). We investigated progressive atrophy using a robust approach to volumetric analysis in a relatively large number of TBI patients in the chronic phase, removing the impact of the acute injury. We used a deformation-based morphometry technique to directly model rates of brain atrophy voxelwise (Ashburner *et al.*, 2000; Hua *et al.*, 2009; Leow *et al.*, 2009; Sarro *et al.*, 2016). This provides a particularly robust approach to quantifying the longitudinal atrophy, and has not been previously applied in TBI in comparison to control data. The analysis models the repeated-measures nature of a longitudinal study during image processing, providing greater sensitivity and reducing biases from ‘asymmetric’ imaging analyses (Reuter *et al.*, 2012; Ashburner and Ridgway, 2013). Importantly, we included a longitudinal control group, allowing us to explicitly test whether atrophy rates in the patient group differed from normal ageing.

These improvements in methodology allowed us to sensitively investigate the spatial pattern of atrophy following TBI. We examined cross-sectional differences in grey and white matter volume, then explored longitudinal patterns of atrophy over approximately 1 year, hypothesizing that TBI patients would show greater atrophy compared to healthy controls. We assessed how these changes relate to cognitive impairment and studied the potential impact of focal brain lesions on the results. Furthermore, evidence from animals, computational models, and human post-mortem data show that physical strain at the time of various types of injury is greater in the cortical sulci than gyri (Cloots *et al.*, 2008; Goldstein *et al.*, 2012; Ghajari *et al.*, 2017). Tau pathology is characteristically seen within the sulci in chronic traumatic encephalopathy (CTE), and has been observed after a single TBI (McKee *et al.*, 2013; Stein *et al.*, 2015). Hence, we predicted that progressive atrophy would be greater in sulcal compared to gyral regions. Finally, we assessed volumetric measures as biomarkers for clinical trials for neuroprotection, presenting power calculations in relation to a commonly used criteria (the so-called ‘n80’) of assessing neuroimaging biomarker efficiency in neurodegenerative conditions (Beckett, 2000; Hua *et al.*, 2009, 2010; Gutman *et al.*, 2015).

## Patients and methods

### Participants

The study included 61 patients who had sustained a single moderate-severe TBI [mean age = 41.6, standard deviation (SD) = 12.8, 49 males, 12 females], all with complete MRI assessment at baseline and follow-up after ˜1 year (median interval = 13.1 months, range = 5.2–25.3). Baseline MRI took place a median of 11.7 months post-injury (range 1.5–562.8). Longitudinal data were acquired from 32 healthy controls (mean age = 34.2, SD = 10.3, 18 males, 14 females), for whom the median interval between baseline and follow-up was 12.7 months (range 8.9–45.1). Further participant details are in Table 1.

**Table 1:** Demographic and clinical characteristics of moderate-severe TBI patients and healthy controls

	TBI patients	Controls	Group comparison
*n*	61	32	–
Age, years, mean ± SD	41.55 ± 12.77	34.22 ± 10.29	*P* = 0.004
Sex, *n*, male/female	49/12	18/14	*P* = 0.027
Interval between scans, months, median [IQR]	13.08 [9.00–15.24]	12.72 [12.09–14.22]	*P* = 0.13
Scanner system, *n*, Philips/Siemens	36/25	13/19	*P* = 0.14
Time since injury, months, median [IQR]	11.71 [4.53–25.21]	–	–
Post traumatic amnesia, *n* (%)	56 (91.8)	–	–
Presence of focal lesions, *n* (%)	41 (67.2)	–	–
Presence of microbleeds, *n* (%)	29 (47.5)	–	–
Lowest recorded GCS score, mean ± SD	8.36 ± 4.92	–	–
Cause of injury, *n*			
Road traffic accident	28	–	–
Violence/assault	15	–	–
Incidental fall	15	–	–
Sport	2	–	–
Unknown	1	–	–

Acute GCS scores were available in *n* = 28 TBI patients.

GCS = Glasgow Coma Scale; IQR = interquartile range.

Recruitment of TBI patients was coordinated through out-patient clinics where individuals were being treated for persistent neurological complaints. The data reported are a combination of a prospective longitudinal study of outcomes after chronic TBI (*n = *36), supplemented by patients who had been recruited into more than one cross-sectional study (*n = *25). This resulted in data from two different MRI scanners being used, with minor differences between acquisition protocols. Patients’ injury severity was classified according to the Mayo Classification System (Malec *et al.*, 2007) and information regarding cause of injury, lowest acute Glasgow Coma Scale score, presence and duration of post-traumatic amnesia were recorded. Post-traumatic amnesia (PTA) was defined by a combination of objectively recorded assessments of PTA in the post-traumatic period, and the retrospective assessment of memory loss for specific post-traumatic events. The duration of PTA was taken to be the interval between the injury and the patient regaining continuous memory for day-to-day events, which has been shown to correlate highly with prospectively acquired assessment of PTA (McMillan *et al.*, 1996). Hospital records and information from relatives were used to establish landmarks against which memory was judged. The presence of focal lesions and cerebral microbleeds was determined by experienced clinical neuroradiologists who assessed multiple modalities of neuroimaging data acquired at baseline (T_1_-weighted, susceptibility-weighted imaging, FLAIR). Exclusion criteria for the TBI patients were: mild/probable injury severity, prior significant TBI, prior psychiatric or neurological illness, current or previous drug or alcohol abuse, MRI contraindication. Ninety patients were recruited into the longitudinal study. Seventeen mild (probable) or symptomatic (possible) patients from this group were not included in the analysis. A further nine patients were excluded following initial scanning for the following reasons that became apparent after the baseline study: premorbid epilepsy, brain tumour identified on MRI (*n = *3), extensive brain injury that precluded accurate image analysis, preceding psychiatric diagnoses, (*n = *3) and drug abuse. The rate of loss to follow-up i.e. a failure to return or reassessment was ∼35%.

Healthy controls were screened according to the same criteria, with the additional requirement of having no history of significant TBI (i.e. no head injury resulting in loss of consciousness or hospital admission). Recruitment of controls was conducted via the local imaging research facilities at Hammersmith Hospital and included data pooled from various on-going studies that used the matching imaging protocols. All participants provided written informed consent and the Hammersmith, Queen Charlotte’s and Chelsea Research Ethics Committee approved the study.

### Neuropsychological testing

The current sample included a proportion of TBI patients involved in our previous cross-sectional studies (Bonnelle *et al.*, 2011, 2012; Kinnunen *et al.*, 2011; Hellyer *et al.*, 2013; Fagerholm *et al.*, 2015). As part of these studies, a standardized battery of neuropsychological tests was administered, as previously documented (Kinnunen *et al.*, 2011). Based on these previous publications, a limited set of neuropsychological tests were analysed in the current study, to reduce multiple testing issues. This selection was based on our previous work demonstrating sensitivity to TBI-related alterations to brain structure. The following tests were assessed: Trail Making Test ‘B minus A’ (executive function), the People test total recall (subsection of Doors and People assessment) (memory), the Choice Reaction Task median reaction time (information processing speed) and Wechsler Abbreviated Scale for Intelligence (WASI) Similarities subscale (intellectual ability). Tests were administered at both baseline and follow-up, although a number of TBI patients did not complete the follow-up (Table 2).

**Table 2:** Longitudinal neuropsychological assessment and relationships with Jacobian determinant measures in TBI patients

	Trail Making Task B−A (s)	People Test (total recall)	WASI similarities (score)	Choice Reaction Task median reaction time (ms)
**Baseline assessment**				
*n*	57	57	36	47
Mean ± SD	37.9 ± 29.2	23.5 ± 7.7	36.7 ± 5.0	497 ± 102
Grey matter Jacobian determinant	ρ = 0.09, *P = *0.49	ρ = 0.14, *P = *0.27	ρ = 0.36, *P = *0.03*	ρ = 0.01, *P = *0.94
White matter Jacobian determinant	ρ = 0.01, *P = *0.92	ρ = 0.14, *P = *0.29	ρ = 0.48, *P = *0.003**	ρ = −0.11, *P = *0.44
**Follow-up assessment**				
*n*	44	44	23	17
Mean ± SD	33.1 ± 1	25.6 ± 8.8	34.7 ± 5.9	541 ± 112
Grey matter Jacobian determinant	ρ = 0.07, *P* = 0.66	ρ = 0.51, *P* 0.0004**	ρ = −0.06, *P* = 0.78	ρ = 0.43, *P* = 0.09
White matter Jacobian determinant	ρ = −0.10, *P* = 0.52	ρ = 0.48, *P* = 0.0008**	ρ = 0.04, *P* = 0.84	ρ = 0.25, *P* = 0.34
**Longitudinal change**				
*n*	41	41	22	16
Mean ± SD	−1.3 ± 29.1	2.0 ± 8.2	−0.05 ± 6.7	1.6 ± 163.4
Grey matter Jacobian determinant	ρ = 0.01, *P* = 0.98	ρ = 0.36, *P* = 0.02*	ρ = −0.19, *P* = 0.39	ρ = 0.37, *P* = 0.16
White matter Jacobian determinant	ρ = 0.10, *P* = 0.51	ρ = 0.34, *P* = 0.03*	ρ = −0.26, *P* = 0.25	ρ = 0.32, *P* = 0.22

*Significance at *P* < 0.05. **Significance after FDR correction for 12 independent comparisons. All statistical tests reported are Spearman’s rank order correlations (ρ) and corresponding *P*-value. WASI = Wechsler Abbreviated Scale for Intelligence. *n* maximum was 61 for all assessments.

### MRI acquisition

High-resolution structural T_1_-weighted images were acquired at 3 T, on two separate MRI scanners. These were a Philips 3 T Achieva (Philips Medical Systems) and Siemens 3 T Verio system (Siemens Healthcare). The proportion of patients and controls investigated on each scanner were similar (Philips/Siemens: TBI patients *n = *36/25, controls *n = *13/19, *χ*^2^ = 2.16, df = 1, *P* = 0.14; Table 1). Importantly, subjects were always scanned at baseline and follow-up on the same system. A Philips T_1_-FE sequence was acquired with voxel dimensions of 1.2 × 0.9375 × 0.9375 mm. Siemens MPRAGE sequences were with voxel dimensions of 1 mm^3^. Full MRI acquisition details are in the Supplementary material. Focal cerebral lesions were detected in *n = *41 TBI patients.

### Neuroimaging processing

**Figure 1:**
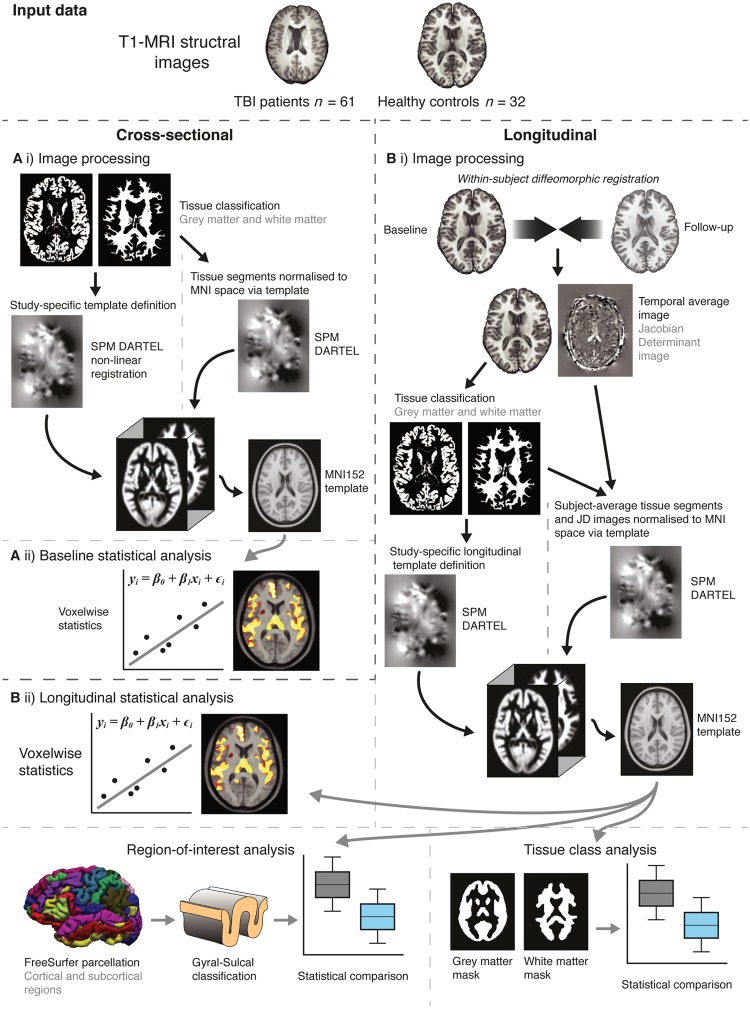
**Overview of study methods.** [**A**(**i**)] Initial processing used SPM to segment T_1_ images into grey and white matter probability maps. A study-specific template was defined based on 40 randomly selected participants (20 TBI patients, 20 controls), using DARTEL registration for non-linear spatial normalization. The template was then affine registered to MNI152 space. All images were then normalized (smoothed by 8 mm and modulated) to MNI152 space via the study template. [**A**(**ii**)] Statistical analysis was carried out voxelwise across the normalized grey and white matter images, using Randomise (FSL) with 5000 permutations using the threshold-free cluster enhancement, adjusting for age and intracranial volume. [**B**(**i**)] Image processing entailed an initial symmetric within-subject registration for each subject’s baseline and follow-up images. This generated a within-subject ‘temporal average’ and Jacobian determinant image, representing the voxelwise spatial expansion and contraction necessary to match baseline and follow-up images. Average images were then segmented into grey and white matter. A random selection of 20 TBI patients and 20 controls was used to define a study-specific longitudinal template with DARTEL, which was then affine registered MNI152 space. Individual average images and Jacobian determinant images were then normalized (smoothed by 8 mm and modulated) to MNI152 space via the longitudinal template. [**B**(**ii**)] Longitudinal analysis included voxelwise group comparisons using Randomise, region of interest analysis based on FreeSurfer (Destriuex) atlas regions and tissue class (i.e. grey and white matter) analysis.

Neuroimaging analysis consisted of two independent comparisons between TBI patients and controls; a cross-sectional comparison of brain structure at baseline and a longitudinal comparison of changes in brain structure over ˜1 year (Fig. 1). Full details are included in the Supplementary material.

In brief, cross-sectional analysis entailed using a standard voxel-based morphometry pipeline (SPM12, University College London, www.fil.ion.ucl.ac.uk/spm). Longitudinal analysis also used SPM12 (Ashburner and Ridgway, 2013), though here the two images from each subject were co-registered to create a Jacobian determinant image that corresponds to the amount of contraction or expansion each voxel undergoes during the study period. These Jacobian determinant values are weighted by the exact inter-scan interval to control for variability due to differing time between scans. Image normalization was then conducted using the ‘temporal’ average images.

**S**ummary measures of subject-space Jacobian determinant images were obtained using grey and white matter tissue masks. Annualized atrophy rates were also calculated as follows:

(1) $$annualized\;atrophy\;rate\;\%=\frac{100\;\times \;\left[\frac{follow‐up\;volume-baseline\;volume}{baseline\;volume}\right]}{interval\;(years)}$$

For region of interest analysis, FreeSurfer (v5.3 http://surfer.nmr.mgh.harvard.edu) cortical and subcortical parcellations of the temporal-average images were obtained for each subject. These Destrieux atlas (Destrieux, 2010) parcellations included 58 gryal and 62 sulcal regions of interest.

### Statistical analysis

Voxelwise statistical analysis used FSL Randomise software (Winkler *et al.*, 2014) for cross-sectional and longitudinal analyses. Group comparisons between TBI patients and controls used the general linear model, with age, sex, scanner type (Philips or Siemens) and intracranial volume as covariates. Multiple testing correction used 10 000 permutations and threshold-free cluster enhancement to generate statistically-corrected voxelwise *P*-values for each contrast, where corrected *P* < 0.05 was considered significant. As the presence of lesions may influence the image processing, the cross-sectional and longitudinal analyses were repeated excluding all TBI patients with a visible lesion (*n = *41).

Analysis of relationships between tissue or region of interest-based Jacobian determinant values or brain volumes with demographic, neuropsychological and clinical measures were conducted using R (www.R-project.org). Bivariate correlations were conducted using Pearson’s r or Spearman ρ tests where appropriate. Main effects of categorical variables (e.g. experimental group, cause of injury) on continuous outcome variables (e.g. mean Jacobian determinant values for grey matter) were tested using linear regression models and included covariates: age, sex, intracranial volume and scanner. To assess the relationship between cognitive function and atrophy, correlations between baseline, follow-up and longitudinal changes in neuropsychological scores, and grey and white matter Jacobian determinant values were conducted. Multiple comparison correction, using the false discovery rate (FDR), was applied across the four neuropsychological tests and visits (baseline, follow-up and longitudinal change), but not tissue type. Longitudinal changes in neuropsychological scores were assessed using mixed-effects models. Hierarchical partitioning analysis (Chevan and Sutherland, 1991) was carried out using the R package ‘hier.part’. This was done to determine the statistically independent influence of different predictor variables, controlling for potentially important confounding effects. To investigate within-subject effects of cortical region of interest classification (i.e. gyrus or sulcus) linear mixed-effects models were used. These models included a random intercept to estimate variance due to between-subject differences in mean atrophy and a random slope to model potentially differing effects of group on region of interest. Models with and without group × region of interest interaction terms were compared to examine interaction effects. Analyses were also re-run excluding TBI patients with lesions.

#### Power calculations

We performed power calculations to assess the utility of different volumetric measures as end-points in clinical trials of neuroprotective treatments following TBI. Effect sizes were calculated using Cohen’s *d* (i.e. group difference in means divided by the pooled standard deviation). Sample size estimates were calculated using the formula from Cash *et al.* (2015):

(2) $$N=2\;\times \;{\left[\frac{1.96\;+\;0.842}{TE\;\times \;ES}\right]}^{2}$$

Where *TE* = treatment effectiveness (e.g. 25% reduction in atrophy rates = 0.25) and *ES* = effect size (e.g. group differences in annualized atrophy rates characterized by Cohen’s *d*). As in the n80 criteria for assessing the efficiency of neurodegenerative biomarkers (Hua *et al.*, 2009), this approach provides sample size estimates *N* for 80% power to detect a change in atrophy rates in cases relative to controls (significance level α = 0.05).

## Results

### Lower cross-sectional brain volumes in patients with traumatic brain injury

**Figure 2:**
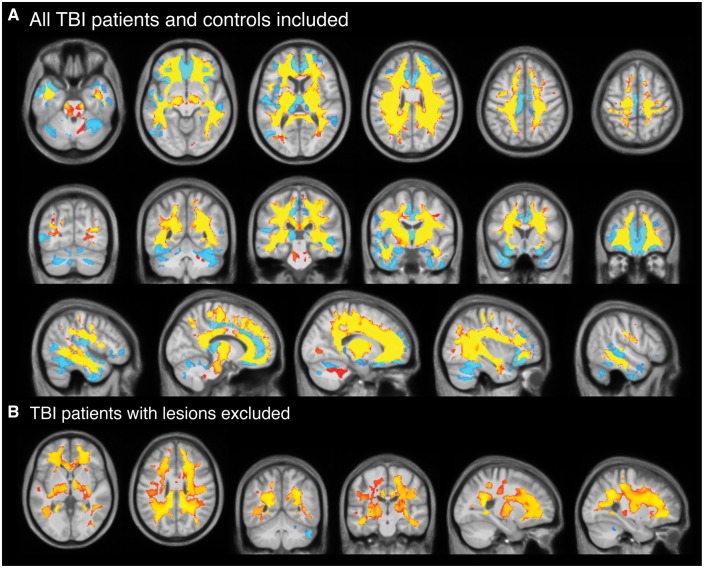
**Cross-sectional voxelwise comparison of brain volume in TBI patients and healthy controls.** (**A**) Voxels showing significantly (corrected *P* < 0.05) lower grey matter (light blue) and white matter (yellow-red) volumes in TBI patients compared to controls at baseline, corrected for multiple comparisons using 10 000 permutations. Slices displayed are axial, coronal and sagittal and overlaid on the study template image. (**B**) Lower grey and white matter volumes in lesion-free TBI patients (*n = *20) compared to controls at baseline, using the same contrast. TBI patients with lesions (*n = *41) were excluded.

At baseline, voxel-based morphometry analysis showed widespread regions of significantly lower grey and white matter volume in TBI patients, compared to controls (Fig. 2A). For grey matter, this included bilateral frontal, temporal and occipital cortical areas, insula cortex, cerebellum and subcortical nuclei (thalamus, hippocampus and amygdala). For white matter, multiple regions showed significantly lower volume in TBI patients, including key areas such as the corpus callosum, corona radiata, internal capsule and brainstem. There were no voxels with significantly greater volume in TBI patients compared to controls.

When removing TBI patients with focal lesions (*n = *41), the extent of grey matter reduced substantially, only remaining significant in the cerebellum, while white matter changes remained visually similar (Fig. 2B). There were no significant differences in the lowest recorded Glasgow Coma Scale (GCS), the presence of microbleeds, the occurrence of PTA, nor on any of the neuropsychological assessments between patients with and without focal lesions.

### Longitudinal brain volume reductions in patients with traumatic brain injury

**Figure 3:**
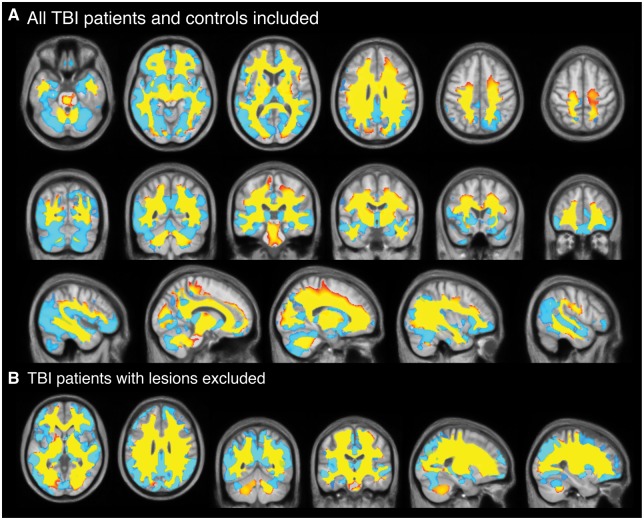
**Longitudinal comparison of voxelwise volume reductions TBI patients and controls.** (**A**) Voxels showing significantly (corrected *P* < 0.05) lower Jacobian determinant values in grey matter (light blue) and white matter (yellow-red) regions in TBI patients compared to controls based on longitudinal image processing and corrected for multiple comparisons using 10 000 permutations. Slices displayed are axial, coronal and sagittal and overlaid on the study template image. (**B**) Voxels showing significantly (corrected *P* < 0.05) lower Jacobian determinant values in grey and white matter regions in lesion-free TBI patients (*n* = 20) compared to controls at baseline, using the same contrast. TBI patients with lesions (*n* = 41) were excluded.

**Figure 4:**
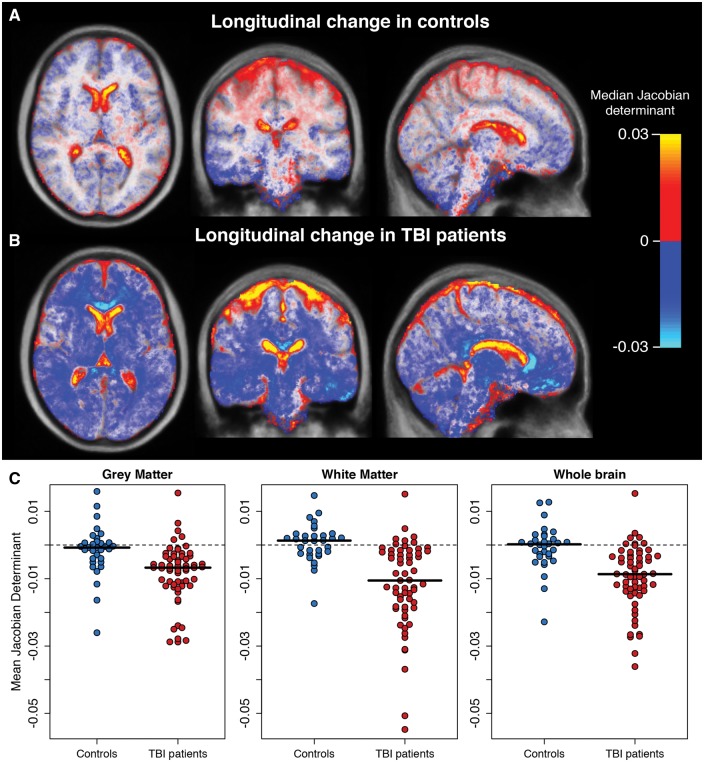
**Jacobian determinant of volumetric change over 12 months in TBI patients and controls.** Median Jacobian determinant images as qualitative illustration of longitudinal change over 1 year in (**A**) healthy controls and (**B**) TBI patients. Hot colours (red–yellow) indicate volumetric increases, while cool colours (blue–light blue) reflect volumetric decreases. Mean Jacobian determinant images are orthogonal slices overlaid on the longitudinal study template. (**C**) Grouped scatterplots representing the mean Jacobian determinant values in both healthy controls and TBI patients, when averaging across grey and white matter regions or the whole brain.

Using annualized estimates (i.e. percentage volume difference over 1 year), TBI patients lost a mean of 1.55% (SD = 2.19) of grey matter volume, 1.49% (SD = 2.20) of white matter volume or 1.51% (SD = 1.60) of whole brain volume. Healthy controls lost 0.55% ± 1.13 of grey matter volume, gained 0.26% ± 1.11 of white matter volume; equating to a 0.22% ± 0.83 reduction in whole brain volume over the same period. Significantly greater voxelwise volume reductions over 1 year, quantified by Jacobian determinant images, were evident in grey matter and extensive white matter regions in TBI patients, compared to controls (Fig. 3A). For grey matter, the frontal, temporal, occipital and insula cortices all showed significant volume reductions bilaterally, as did multiple subcortical nuclei (thalamus, amygdala, hippocampus, caudate, putamen) and the cerebellum. Generally, the grey matter changes were more inferior, with superior areas showing fewer significant differences between groups. For white matter, extensive differences in Jacobian determinants were evident in TBI patients, equating to 85.4% of voxels tested, which included the majority of the white matter pathways. No grey matter or white matter voxels showed significant Jacobian determinant increases in TBI patients compared to controls. The widespread nature of the brain volume decreases in TBI patients, relative to controls, was further illustrated by averaging Jacobian determinant values for each group (Fig. 4A and B). Removing TBI patients with focal lesions (*n = *41) from the analysis resulted in patterns of atrophy that were nearly identical to the whole group of TBI patients. The spatial pattern of significantly lower Jacobian determinant values in TBI patients without focal lesions were very similar to the whole TBI group for both grey and white matter changes (Fig. 3B).

### Longitudinal volume changes in relation to clinical characteristics of traumatic brain injury

To investigate patterns of volumetric decreases seen in TBI patients further, tissue-specific (i.e. grey matter or white matter) and whole-brain mean Jacobian determinant was calculated for each participant (Fig. 4C). Mean grey matter Jacobian determinant was significantly lower in TBI patients compared to controls [*b* = −0.007, standard error (SE) = 0.002, *t* = −3.56, *P* = 0.0008], as was mean white matter Jacobian determinant (*b* = −0.01, SE = 0.003, t = −4.38, *P* = 2.6 × 10^−5^). The model was adjusted for age, sex, intracranial volume and scanner. Hierarchical partitioning of variance was used to establish unique contributions of different predictor variables, the influence of scanner type on Jacobian determinant values was explored further. Independent R^2^ values explaining grey matter Jacobian determinant variance were as follows: group = 12.2%, age = 0.7%, sex = 0.3%, scanner = 9.2% (total R^2^ = 22.3%). For white matter Jacobian determinant values, the R^2^ values were: group = 19.8%, age = 0.4%, sex = 0.6%, scanner = 9.0% (total R^2^ = 27.6%). The shared variance between group and other predictors was R^2^ = 1.0% for grey matter and R^2^ = 3.2% for white matter.

Using mean grey and white matter Jacobian determinant as summary measures of atrophy per tissue type, relationships with clinical characteristics of TBI were investigated. Mean grey and white matter Jacobian determinant were not significantly related to time since injury (accounting for age at baseline, grey matter *P* = 0.51; white matter *P* = 0.80). Nor were Jacobian determinant values associated with the presence of post-traumatic amnesia (grey matter *P* = 0.48, white matter *P* = 0.98) or cerebral microbleeds (grey matter *P* = 0.23; white matter *P* = 0.89). There was no difference in Jacobian determinant values in TBI patients with lesions compared to those without (grey matter *P* = 0.48, white matter *P* = 0.31). Lesion extent (i.e. volume) was not related to Jacobian determinant values (grey matter *P* = 0.50; white matter *P* = 0.21). Lowest recorded GCS scores were available in a subset of TBI patients (*n = *28) and these values were not associated with mean grey matter (*P* = 0.29) or white matter (*P* = 0.75) Jacobian determinant values. Cause of injury (i.e. road-traffic accident, assault, fall, sporting injury) category did not influence mean Jacobian determinant values for grey matter (*P* = 0.28) or white matter (*P* = 0.48).

### Longitudinal volume changes and neuropsychological performance

Neuropsychological test results are shown in Table 2. Longitudinal changes in performance were not significant for any test (*P* > 0.14), with mean test scores remaining generally consistent between visits. However, when comparing Jacobian determinant values with neuropsychological test scores, significant relationships (FDR corrected for 12 tests) were evident at baseline for WASI Similarities and white matter (ρ = 0.48, *P* = 0.003), and at follow-up for People test total recall for both grey matter (ρ = 0.51, *P* = 0.0004) and white matter (ρ = 0.48, *P* = 0.0008), with greater atrophy associated with poorer performance. Significant associations were also observed for baseline grey matter mean Jacobian determinant and Similarities (ρ = 0.36, *P* = 0.03) and longitudinal change in People and Doors total recall, both grey matter (ρ = 0.36, *P* = 0.02) and white matter (ρ = 0.24, *P* = 0.03), but these did not survive FDR correction for multiple comparisons.

### Greater atrophy is seen in sulcal regions of the cortex after traumatic brain injury

**Figure 5:**
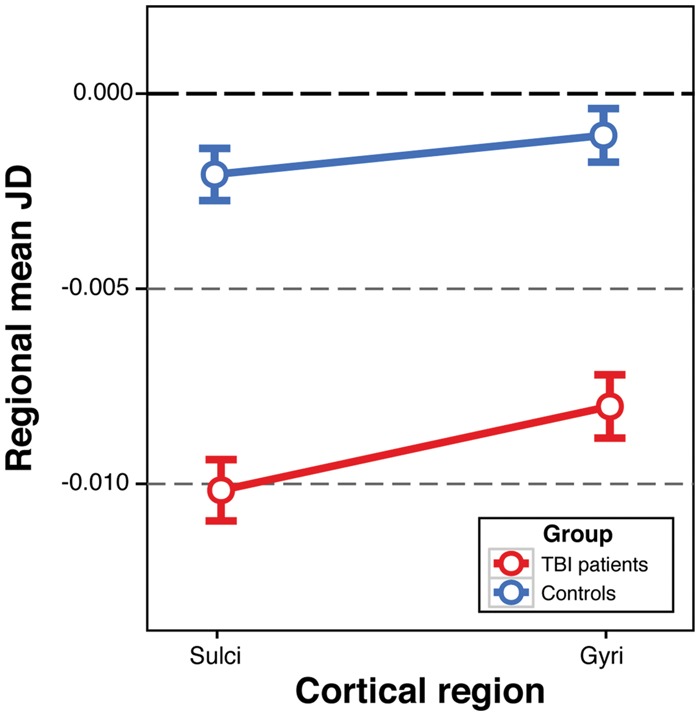
**Atrophy according to cortical region classification in TBI patients and controls.** Plot showing the mean and standard error of Jacobian determinant (JD) values in TBI patients (red) and controls (blue) according to classification of cortical grey matter regions of interest as either sulci or gyri. Differences between TBI patients and controls are observed in both sulci and gyri, however the interaction between group and region of interest was significant (*b* = 0.001, SE = 0.0006, *t* = 2.08, *P* = 0.037), reflecting greater atrophy in the sulci of TBI patients compared to the gyri.

Neuropathology associated with chronic traumatic encephalopathy at post-mortem is seen disproportionately within the sulci (McKee *et al.*, 2013), which is also the site of high biomechanical strain in various types of injury (Ghajari *et al.*, 2017). Hence, we tested whether atrophy was more pronounced in these regions; our analysis indicated that sulcal regions show greater atrophy after TBI. Main effects of both group (*b* = −0.008, SE = 0.002, *t* = −3.80, *P* = 0.0003) and region of interest (gyrus or sulcus) were significant (*b* = 0.002, SE = 0.0003, *t* = 6.67, *P* = 2.6 × 10^−11^) in a linear mixed-effects model. An interaction model included a group × region of interest term, which was significant (*b* = 0.001, SE = 0.0006, *t* = 2.08, *P* = 0.037). Plotting these effects illustrated the greater atrophy in sulci compared to gyri in TBI patients (Fig. 5). When excluding TBI patients with lesions from the analysis, both the main effects of group (*P* = 0.002) and region of interest (*P* = 1.5 × 10^−14^), and the interaction between group and region of interest, remained significant (*P* = 4.7 × 10^−8^). There was no interaction between injury cause and region of interest (*P* = 0.10).

### Jacobian determinants effect sizes and sample size calculations

**Figure 6:**
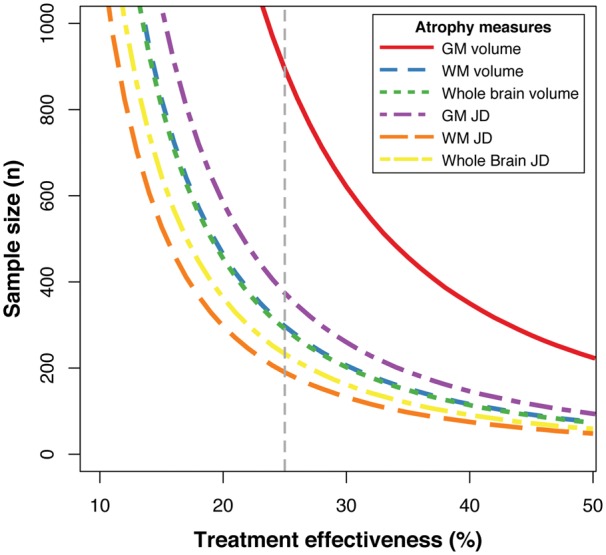
**Clinical trial sample size requirements based on brain atrophy measures.** Sample size requirements for a placebo-controlled clinical trial of a treatment aimed at reducing atrophy in cases relative to controls. Sample sizes are plotted against potential treatment effectiveness. Coloured lines depict this relationship for different atrophy measures, either volumetric or deformation-based (i.e. Jacobian determinant values), using either grey matter, white matter or whole brain volume. Grey dashed vertical line indicates the effectiveness level used in the sample size calculations presented in the ‘Results’ section (i.e. 25% effectiveness). GM = grey matter; JD = Jacobian determinant; WM = white matter.

Next we compared the sample sizes needed to detect intervention effects using different T_1_-MRI measurements (Fig. 6). Longitudinal volumetric group differences in annualized volume change (normalized for baseline volume) were: grey matter Cohen’s *d* = 0.53 [95% confidence interval (CI) 0.09–0.97], white matter *d* = 0.92 (95% CI 0.47–1.38]) and whole brain *d* = 0.93 (95% CI 0.48–1.38). For mean Jacobian determinant metrics, group difference effect sizes were: grey matter *d* = 0.82 (95% CI 0.37–1.27), white matter *d* = 1.15 (95% CI 0.68–1.61) and whole brain *d* = 1.04 (95% CI 0.58–1.50). The required sample size per treatment arm of a placebo-controlled trial was estimated, assuming 80% power to detect a significant effect (α = 0.05), and varied as a function of putative treatment effectiveness. As an example, in trials of drug intervention in Alzheimer’s disease a 25% effectiveness in reducing atrophy rates relative to healthy individuals is commonly used (Hua *et al.*, 2009; Cash *et al.*, 2015). This magnitude of treatment effectiveness would require the following sample sizes (per group) for different imaging metrics: grey matter volume, *n = *894; white matter volume, *n = *297; whole brain volume, *n = *291; grey matter Jacobian determinant, *n = *374; white matter Jacobian determinant, *n = *190; whole brain Jacobian determinant, *n = *233. The required sample size varies as a function of treatment effectiveness (Fig. 6). For treatment effects of 25% or below, the added-value of using the Jacobian determinant method to reduce sample size is clear, although as the effectiveness reaches 50% the benefits diminish as the required sample sizes converge.

## Discussion

We observed widespread brain tissue volume loss over a 1-year period in the chronic phase after moderate-severe TBI. This atrophy was unrelated to the time since a patient’s injury, indicating that progressive loss of brain volume can occur from several months to many years after injury. Importantly, we confirmed this differs from normal ageing as there was minimal volume change in a healthy control group. The marked TBI-related atrophy affects multiple grey and white matter areas, with effects that are most prominent in the white matter where the majority of tracts were affected, which may be a result of diffusion axonal injury. While many cerebral grey matter regions also showed atrophy, the effect was greater in sulcal compared to gyral regions. Tau pathology is characteristically seen in the depths of cortical sulci in CTE (McKee *et al.*, 2013) and similar pathology can be seen after single TBI (Johnson *et al.*, 2012). As tau pathology is associated with atrophy (Rohrer *et al.*, 2011; Holmes *et al.*, 2016; LaPoint *et al.*, 2017; Maass *et al.*, 2017), mapping the location of atrophy might be informative in delineating neuropathology after TBI.

Our study builds on previous work showing progressive atrophy after TBI (Trivedi *et al.*, 2007; Bendlin *et al.*, 2008; Green *et al.*, 2014). We extended these studies by investigating a larger TBI patient sample in the chronic phase after injury and using optimal approaches to image analysis. We studied a healthy control group longitudinally, which allowed accurate assessment of post-traumatic atrophy in the context of healthy ageing. Within-subject atrophy effects were modelled at the image processing stage with advanced registration procedures (Ashburner and Ridgway, 2013). This provided a robust experimental approach allowing us to examine atrophy patterns in relation to various important features specific to TBI, including the impact of focal lesions.

Neurodegenerative conditions have distinct patterns of atrophy that are informative diagnostically. For example, the presence of progressive hippocampal atrophy is a marker of Alzheimer’s disease (Scahill *et al.*, 2002). Hence, tracking atrophy after either mild or moderate-severe, single or repetitive TBI could provide diagnostic information about the presence and type of post-traumatic neurodegenerative pathology, in particular the development of CTE. Here, greater atrophy was seen in cortical sulci compared to gyri, reminiscent of the location of CTE pathology (McKee *et al.*, 2013). Our computational modelling has shown that high biomechanical strains are present at the time of similar types of single head injuries within sulcal regions (Ghajari *et al.*, 2017). This distribution of biomechanical forces is likely to result in greater acute damage and subsequently more neurodegeneration, which as we have demonstrated can be tracked using longitudinal MRI.

The magnitude of longitudinal changes was not merely driven by baseline brain volumes, nor the extent of localized lesions. While our cross-sectional results broadly replicate previous voxel-based morphometry studies of TBI patients (Gale *et al.*, 2005; Vannorsdall *et al.*, 2010; Messé *et al.*, 2011), our analysis indicates that the baseline grey matter volume difference were, to an extent, driven by the presence of focal lesions. This highlights the importance of accounting for post-traumatic lesions during image processing, something that is commonly overlooked by previous studies. In contrast, white matter volume differences were not substantially affected by the presence of focal lesions, perhaps because lesions predominantly affected the grey matter. Importantly, the longitudinal changes identified in both grey and white matter were largely unaffected by the presence or absence of lesions, showing that the unbiased within-subject longitudinal registration procedure we used is robust to the presence of lesions when assessing brain atrophy. Nevertheless, future studies could investigate how the presence of focal lesions influences atrophy at later time points post-injury.

We show that longitudinal changes in volumetric neuroimaging measures are a sensitive way to assess post-traumatic white matter pathology. Both our cross-sectional and longitudinal analyses indicated that white matter was strongly affected after TBI, in line with historical and contemporary research (Bigler, 2013). Much recent work in the TBI field, including in our own laboratory, has focused on the use of diffusion MRI to investigate white matter after TBI (Kinnunen *et al.*, 2011; Bonnelle *et al.*, 2012; Hulkower *et al.*, 2013). While diffusion tensor imaging (and related diffusion-MRI methods) may be sensitive to a broader range of pathological effects than T_1_-MRI, the neurobiological processes underlying changes in fractional anisotropy and other related diffusivity measures are still contentious. Diffusion tensor imaging is also hampered by lower resolution, increased acquisition time and poorer test re-test reliability relative to T_1_-MRI (Landman *et al.*, 2011). As T_1_-MRI is routinely collected in clinical settings and is easier to combine across scanners, it is in many ways more appropriate for large-scale studies investigating white matter pathology after TBI (Johnson *et al.*, 2013).

Efforts to develop new treatments for TBI-related neurodegeneration might usefully follow examples of other neurodegenerative conditions (e.g. Alzheimer’s and Huntington’s diseases) in using neuroimaging biomarkers to aid in clinical trial design. Our results also show that summarizing Jacobian determinants values leads to greater sensitivity to atrophy compared to averaged change in tissue volume, a result previously reported in Alzheimer’s disease (Hua *et al.*, 2016). This is likely to be because Jacobian determinant images directly map change at individual voxels, whereas volumetric methods are strongly driven by changes at structural boundaries, such as tissue–CSF interfaces. Hence, regional volume measures may be insensitive to subtle changes within brain structures (Leow *et al.*, 2006).

The comparative sensitivity of atrophy measures is important because this influences the sample sizes needed to detect experimental or therapeutic effects. For example, structural MRI atrophy measures have been commonly used as outcome measures in clinical trials of neurodegenerative disease treatments (Doody *et al.*, 2014; Salloway *et al.*, 2014). This approach has yet to be applied in TBI but there are a number of reasons it is attractive for clinical trials of post-traumatic neurodegeneration. For example: (i) atrophy has a strong face-validity as the progressive loss of brain tissue is closely related to neurodegeneration that is itself a negative long-term effect of TBI; (ii) it is related to cognitive impairments in our study and others, as well as poorer functional outcomes (Sidaros *et al.*, 2009; Ross *et al.*, 2012; Brezova *et al.*, 2014); (iii) it is substantially more affected by TBI than other potential confounds such as age and sex (∼20% versus ∼0.5%); and (iv) high statistical reliability and sensitivity allow for smaller sample sizes.

As shown by our sample size estimates, using Jacobian determinant measures of white matter atrophy the size per treatment arm could be reduced to *n = *190, compared to *n = *291 using whole-brain volume, to detect a 25% reduction in brain atrophy. The magnitude of this treatment effect is somewhat arbitrary, but has been used as a basis for criteria used to judge the efficiency of neuroimaging biomarkers for assessing neurodegeneration (e.g. n80 as per Hua *et al.*, 2009). It is uncertain whether this is an optimal criterion for assessing potential treatments following TBI, but extrapolating from the Alzheimer’s disease field seems reasonable as the rates of atrophy observed are similar (Sluimer *et al.*, 2008). Using this value, our calculations indicate that clinical trials of neuroprotective interventions could feasibly use atrophy measures derived from serial T_1_ as a surrogate marker of neurodegeneration after TBI in relatively small clinical trials. The cost of MRI is an important consideration. However, the use of volumetric T_1_ is likely to be cost effective as it can be rapidly acquired, data can be mixed across centres and there is likely to be a large benefit in informing the design and likely utility of larger phase III studies that use conventional clinical end-points.

Longitudinal atrophy was related at baseline to a measure of verbal reasoning (WASI Similarities) and at follow-up to a measure of memory (People subtest of the Doors and People task). This suggests that greater atrophy after TBI may be associated with poorer cognitive performance in certain domains. Interestingly, atrophy also related to change in memory performance, though this did not remain significant after correction for multiple comparisons. This finding concurs with the study of Zhou *et al.* (2013), who reported change in verbal learning, memory performance and information processing speed related to greater reductions in anterior cingulate volume in mild TBI patients. If progressive loss of brain volume is linked to on-going deterioration in cognitive performance, then this indicates that neuroimaging-based atrophy measures could be used as a reliable way to predict individuals at risk of cognitive decline after TBI. However, this preliminary finding requires further investigation using larger sample sizes, studied at more frequent testing after their injury.

Generally, cognitive performance was stable across the study period. While there are some relationships between neuropsychological tests and atrophy, the absence of stronger relationships is notable given the pronounced atrophy observed. This has been shown in other studies (Sidaros *et al.*, 2009; Tomaiuolo *et al.*, 2012; Zhou *et al.*, 2013) and there are a number of possible reasons for this. One explanation is that redundancy and/or functional compensation exists within brain networks that support cognitive function, meaning that neurodegeneration could occur within a network without obvious impact on cognitive performance. This has been proposed as an explanation for atrophy preceding the overt manifestation of neurodegenerative disease including Alzheimer’s and Huntington’s diseases by many years (Stern, 2012; Bonner-Jackson *et al.*, 2013). The natural history of post-traumatic neurodegeneration and its clinical correlates are in some ways more complex than other neurodegenerative conditions, as acute effects of the initial injury and their subsequent recovery accompany the progressive neurodegeneration we have observed. The interaction between these distinct processes will be an important focus for future research. The experimental power to detect a relationship between atrophy and cognitive function is likely to be higher in a sample with a more uniform time since injury, but it would be particularly informative to perform longitudinal studies on large numbers of patients with multiple assessment points after injury, as this would allow the impact of distinct pathophysiological processes on atrophy to be delineated. Future studies of this type would be able to clarify the time course of post-traumatic neurodegeneration and its relationship to cognitive impairment at different times since injury.

We also failed to find a relationship between atrophy and a number of other clinical measures of TBI severity. Previous studies report relationships with longitudinal atrophy and loss of consciousness (MacKenzie *et al.*, 2002), duration of coma (Trivedi *et al.*, 2007; Sidaros *et al.*, 2009) or post-traumatic amnesia (Sidaros *et al.*, 2009; Brezova *et al.*, 2014), and functional outcome (Ding *et al.*, 2008; Sidaros *et al.*, 2009; Warner *et al.*, 2010). We were unable to replicate these findings, and found no relationships between atrophy and clinical parameters commonly used to characterize TBI, including the presence of post-traumatic amnesia, focal lesions, microbleeds or GCS score. This could reflect the high variability in clinical progression following injury, which is not necessarily predicted by these acute measures. Importantly, our analysis is differentiated from these previous studies by a greater time between injury and baseline scan (median = 11.7 months), compared to less than 6 months. Therefore, the relevance of these acute factors to brain structure may decrease as time passes and the pathophysiology of the injury evolves.

There are some limitations to the current study. First, our analysis was carried out at two time points, restricting us to assume linear trajectories of brain structure changes. In reality, the pattern of atrophy may be non-linear over time, with contrasting periods of accelerating or plateauing atrophy. Hence, future studies would benefit from more frequent imaging to model such effects. Second, our study merged data acquired on two separate MRI scanner systems. Scanner type can influence derived imaging metrics (Jovicich *et al.*, 2006), which was seen in the hierarchical partitioning results. Importantly, both patients and controls were collected on both scanners and the two groups were reasonably well-balanced across each scanner, allowing scanner effects to be statistically modelled. Participants always had baseline and follow-up scans performed on the same scanner. Moreover, the hierarchical partitioning analysis indicated that although scanner type explained a substantial proportion of variance, the variance according to group was greater than that of scanner. Crucially the group effect was statistically independent of the scanner influence, so any effect of scanner on Jacobian determinant values did not bias the reported group differences. Hence, the use of two scanners did not affect our finding of group differences in atrophy rates, indicating that clinical trial feasibility could be increased by adopting a design with multiple study centres. Another limitation is that the participants in this study are a subset of all moderate/severe TBI patients and there are a number of possible selection biases that might have influenced the results. For example, patients were recruited predominantly from neurology follow-up clinics. They were patients with on-going neurological problems after TBI, which might make it more likely for this group to have progressive neuropathology compare to the whole population of moderate/severe TBI patients. In addition, the rate of loss to follow-up was high, although comparable to other TBI studies, which may have influenced the results. Further studies will be needed to determine the prevalence of increased atrophy after TBI. Neuropsychological test data were missing from some TBI patients at follow-up, which restricted statistical power and potentially more severely impaired individuals were more likely to not complete the testing. Nevertheless, all participants tolerated MRI scans at both visits, so group-level neuroimaging findings were not influenced by this. Also, using only two time points meant that we were unable to model non-linear trajectories in neuropsychological performance, nor did the sample size allow identification of robust clinically-meaningful subgroups. Finally, the groups were not well-matched for sex or age, with the controls being younger, and the interscan interval was not uniform, ranging up to 4 years. However, neither age nor sex were related to Jacobian determinant measures in either group, so this not did influence the results of the study.

To conclude, we found evidence of progressive brain volume loss months and years after a moderate to severe TBI. This atrophy is particularly pronounced in white matter regions. Grey matter regions also show atrophy, particularly the cortical sulci. Initial clinical characteristics and length of time since injury did not relate to the magnitude of atrophy over 1 year. Atrophy levels related to cross-sectional measures of memory and verbal reasoning and a relationship with atrophy and decline in memory performance was seen, before multiple comparison correction. Progressive atrophy is likely to be relevant to longer-term prognosis and is indicative of progressive neurodegeneration, often many years post-injury. The Jacobian determinant approach to quantifying atrophy proved to be more sensitive to volumetric changes than using macroscopic volumes, and is sufficiently sensitive to afford feasible samples size for trialling future treatments aimed at slowing or stopping the progressive loss of brain tissue seen after TBI.

## Supplementary Material

Supplementary Data

## Abbreviation

TBI: traumatic brain injury
