# Supplementary material for: Spatial patterns of progressive brain volume loss after moderate-severe traumatic brain injury
Source: Brain. 2018 Jan 4;141(3):822–36. doi: 10.1093/brain/awx354 (PMC5837530; doi:10.1093/brain/awx354)
Supplement: Supplementary Data [file awx354_brain-2017-00686-file010.pdf]

Supplementary material

Supplementary Methods

MRI acquisition

High-resolution structural T1-weighted images were acquired at 3T, on two separate MRI scanners. These were a Philips 3T Achieva (Philips Medical Systems, Netherlands) and Siemens 3T Verio system (Siemens Healthcare, Germany). The proportion of patients and controls investigated on each scanner were similar (Philips/Siemens: TBI patients N = 36/25, controls N = 13/19, $X^2 = 2.16$, df = 1, P = 0.14, see Table 1). Importantly, subjects were always scanned at baseline and follow-up on the same system. A Philips T1-FE sequence was acquired with the following parameters: repetition time = 9.60 ms; echo time = 4.60ms; flip angle = 8°; field of view = 24 cm x 24 cm; matrix = 208 x 208; number of slices = 150, slice thickness = 1.2 mm; giving final voxel dimensions of 1.2 x 0.9375 x 0.9375 mm. For the Siemens scanner an MPRAGE sequence was acquired with the following parameters: repetition time = 2300 ms; echo time = 2.98 ms; flip angle = 9°; field of view = 25.6 cm x 24 cm; matrix = 256 x 240; number of slices = 160, slice thickness = 1 mm; giving final voxel dimensions of 1 mm^3. Focal cerebral lesions were detected in N = 41 TBI patients.

Neuroimaging processing

Neuroimaging analysis consisted of two independent comparisons between TBI patients and controls; a cross-sectional comparison of brain structure at baseline and a longitudinal comparison of changes in brain structure over approximately one year (see Fig. 1).

Cross sectional analysis

T1-weighted images from TBI patients (N = 61) and controls (N = 32), were analysed using a standard voxel-based morphometry approach (SPM12, University College London, www.fil.ion.ucl.ac.uk/spm). This involved tissue classification into grey and white matter segments, definition of a study-specific template using 40 randomly-selected subjects (20 TBI patients, 20 controls), non-linear registration to this template using DARTEL (Diffeomorphic Anatomical Registration using Exponentiated Lie algebra) and normalisation to Montreal Neurological Institute (MNI)-152 space with resampling to 1.5mm^3 voxels. This was done for both grey and white matter and images were i) smoothed with an 8mm full-width half maximum Gaussian kernel to improve signal-to-noise ratio and reduce the impact of potential mis-registration and ii) modulated to retain information relating to initial head size, enabling voxelwise comparison of brain tissue volumes. Intra-cranial volume estimates were generated during tissue classification. Each voxelwise analysis

was masked to limit the number of voxels included. Masks for grey and white matter were defined by taking the median of smoothed images for all subjects used in generating the template and thresholding this median image at ≥ 0.4.

Longitudinal analysis

Here we used SPM12 longitudinal registration (Ashburner and Ridgway, 2013). This first calculates a ‘temporal average’ image for each subject, by iteratively co-registering the subject’s baseline and follow-up images, from which an image of the Jacobian determinants is generated. A Jacobian determinant represents the amount of geometric warping a voxel undergoes in order to be mapped between baseline and follow-up images; a positive value indicates an increase in size, a negative value indicates a decrease in size. Importantly, the time between scans was used to weight the Jacobian determinants so that they represent consistent estimates of longitudinal change between subjects with differing inter-scan intervals.

The second stage involved segmenting these ‘temporal average’ images into grey and white matter. A randomly-selected subsample was then used to define a study-specific longitudinal template (20 TBI patients, 20 healthy controls), using the same DARTEL procedure as the cross-sectional analysis. Next, grey and white matter tissue segmentation images were multiplied by Jacobian determinant images to give tissue-specific Jacobian determinant images, and these were registered to MNI152 space, via the longitudinal template, using DARTEL. Again smoothing with an 8mm full-width half maximum kernel was used and the data were modulated to preserve tissue amounts. This process resulted in normalised grey and white matter Jacobian determinant images, appropriate for voxelwise statistical analysis. Masks for the voxelwise analysis were defined as per the cross-sectional procedures.

Finally, summary measures of subject-space Jacobian determinant images were obtained. These included the mean Jacobian determinant value across subject-specific grey and white matter tissue masks, which represent tissue-specific measures of volumetric change between baseline and follow-up (Ashburner and Ridgway, 2013). Annualised atrophy rates were also calculated as follows:

$$annualised\ atrophy\ rate\ \% = \frac{100 \times \left[\frac{follow\_up\ volume - baseline\ volume}{baseline\ volume}\right]}{interval\ (years)}$$

To obtain values for ROI analysis, FreeSurfer (v5.3 http://surfer.nmr.mgh.harvard.edu) *recon-all* was used to parcellate the temporal-average images for each subject into cortical and subcortical regions. These 165 parcellations (Destrieux *et al.*, 2010) were overlaid on the Jacobian determinant images

and mean Jacobian determinant values for each ROI calculated per individual. Classification of ROIs was based on cortical location, either gyrus (n = 58) or sulcus (n = 62), as defined by the Destrieux atlas.
